# dsLassoCov: a federated Lasso approach incorporating covariate control

**DOI:** 10.1038/s41598-026-48845-0

**Published:** 2026-04-22

**Authors:** Han Cao, Augusto Anguita-Ruiz, Charline Warembourg, Xavier Escribà-Montagut, Martine Vrijheid, Juan R. Gonzalez, Tim Cadman, Adrià Setó-Llorens, Verena Schneider-Lindner, Daniel Durstewitz, Xavier Basagaña, Emanuel Schwarz

**Affiliations:** 1 Department of Theoretical Neuroscience, Central Institute of Mental Health, Medical Faculty Mannheim, Heidelberg University, Mannheim, Germany; 2 ISGlobal, Barcelona, Spain; 3 Univ Rennes, EHESP, Irset (Institut de recherche en santé, environnement et travail) - UMR_S 1085, Rennes, 35000 France; 4 Department of Anesthesiology and Surgical Intensive Care Medicine, Medical Faculty Mannheim, Heidelberg University, Theodor-Kutzer-Ufer 1-3, 68167 Mannheim, Germany; 5 Hector Institute for Artificial Intelligence in Psychiatry, Central Institute of Mental Health, Medical Faculty Mannheim, Heidelberg University, Mannheim, Germany; 6 Department of Psychiatry and Psychotherapy, Central Institute of Mental Health, Medical Faculty Mannheim, Heidelberg University, Mannheim, Germany; 7 Genomics Coordination Center, Department of Genetics, University Medical Centre Groningen, Groningen, Netherlands; 8 CIBER Epidemiologa y Salud Pública, Madrid, Spain; 9 CIBER de Fisiopatología de la Obesidad y Nutrición (CIBEROBN), Instituto de Salud Carlos III, Madrid, 28029 Spain

**Keywords:** Data integration, Machine learning, Predictive medicine, Statistical methods, Computational science, Computer science, Statistics

## Abstract

Machine learning has been widely adopted in biomedical research, fueled by the increasing availability of data. However, integrating datasets across institutions is challenging due to legal restrictions and data governance complexities. Federated learning allows the direct, privacy preserving training of machine learning models using geographically distributed datasets, but faces the challenge of how to appropriately control for covariate effects. The naive implementation of conventional covariate control methods in federated learning scenarios is often impractical due to the substantial communication costs, particularly with high-dimensional data. To address this issue, we introduce dsLassoCov, a machine learning approach designed to control for covariate effects and allow an efficient training in federated setting. In biomedical analysis, this may support the identification of biomarker candidates while accounting for the effect of confounding. Using simulated data, we demonstrate that dsLassoCov can efficiently and effectively manage confounding effects during model training. In our real-world data analysis, we replicated a large-scale exposome analysis using data from six geographically distinct databases, achieving results consistent with previous studies. By addressing the challenge of covariate control, our proposed approach can accelerate the application of federated learning in large-scale biomedical studies.

**Supplementary Information:**

The online version contains supplementary material available at 10.1038/s41598-026-48845-0.

## Introduction

Big data technologies have been widely adopted in biomedical research, with machine learning (ML) analysis demonstrating significant success in various molecular^1^ and clinical^2^ applications. This success stems from the development of accurate and generalizable models through training on extensive datasets. However, a central challenge in biomedical research is that data sources are often confined to isolated databases within individual institutes or hospitals. These databases often operate under distinct standards and governance structures^3^, limiting the ability to pool data effectively. The impediment to data pooling is also made difficult by policy regulations, such as the General Data Protection Regulation^4^ (GDPR) in the EU, which requires explicit consent from the patients for the transfer of their data^3^. This challenge significantly hampers the generalizability of ML technologies, e.g., deep learning, which heavily relies on access to extensive training data, and becomes more pronounced in data-scarce areas, such as rare disease analysis^5^. As highlighted in a recent review^6^, federated learning emerges as a promising cross-silo ML technology that safely facilitates model learning by leveraging all available data.

Federated learning^7^ (FL) is a distributed ML approach designed to enhance data privacy protection. In the context of cross-silo scenarios, the FL model undergoes training on multiple isolated databases, often geographically dispersed. Each database resides behind the firewall on the server of the corresponding institution, and a network with limited access is established among these distributed databases to enable authorized access. Here, only non-disclosive parameters of models^8^, devoid of sensitive information, are allowed to be exchanged among the database servers. FL provides the advantage of leveraging multiple datasets for ML model training whilst reducing concerns about the disclosure or exposure of individual-level information. This innovative approach addresses important ethical and legal challenges inherent in data analysis in biomedicine. FL has demonstrated significant potential in various domains, including mitigating data imbalance issues through federated Genome-Wide Association Study (GWAS) analysis^9,10^, as well as detecting rare diseases through the federated integration of data cohorts^11^.

Federated Lasso has been widely used for training high-dimentional, geo-distributed data. Early studies^12^ on distributed Lasso demonstrated that sparse linear regression can be solved across multiple data sites without pooling raw data, often through consensus-based optimization such as ADMM. Building upon this foundation, more general communication-efficient frameworks have been proposed for solving L1-regularized empirical risk minimization problems, including methods such as CoCoA^13^, which provide flexible distributed optimization schemes applicable to Lasso and related sparse models. In addition, distributed coordinate descent algorithms have been developed for regularized generalized linear models, further improving scalability in high-dimensional settings^14^. More recently, privacy-preserving and federated extensions of Lasso have been proposed to enable secure multi-institutional learning, including approaches based on federated coordinate descent and secure aggregation protocols^15,16^. These methods demonstrate that high-dimensional sparse modeling can be effectively implemented under data-sharing constraints. However, most existing approaches primarily focus on optimization efficiency, communication cost, or privacy guarantees, and typically assume that confounding effects can be addressed locally or ignored during model training.

In contrast, in non-federated settings, covariate adjustment has long been recognized as a critical step in high-dimensional statistical modeling, particularly in biomedical research. Standard approaches typically incorporate covariates directly into regression models or apply residualization procedures to remove confounding effects prior to feature selection^17^. For example, it is common practice to regress out known confounders (e.g., age, sex, batch effects) from both predictors and outcomes before applying machine learning approach. In high-dimentional setting, the the sparse modeling, such as partialling-out strategies and debiased or double machine learning frameworks, have also been proposed to ensure valid inference under high-dimensional confounding^18,19^. These approaches have demonstrated strong effectiveness in centralized settings, where all data are available for joint modeling. However, such strategies are not directly applicable in federated learning scenarios, where individual-level data cannot be shared across sites, and covariate distributions may differ substantially between cohorts. As a result, there remains a methodological gap in integrating principled covariate control into federated high-dimensional sparse learning.

Given the aforementioned considerations, we introduce dsLassoCov, a federated Lasso approach designed to control confounding effects in high-dimensional settings. The algorithm is derived from the proximal gradient framework, and we provide a formal interpretation of its covariate adjustment mechanism. We further implement and validate dsLassoCov within the DataSHIELD software ecosystem. Using simulated data, we demonstrate that our method achieves improved computational efficiency, higher feature selection accuracy, and lower coefficient estimation error compared with both federated baseline approaches and meta-analysis strategies. Finally, we illustrate its practical utility through applications to multiple real-world exposome datasets from the HELIX (Human Early-Life Exposome) Project^20^.

## Methods

Our implementation of dsLassoCov (dataSHIELD LASSO regression adjusted for covariates) is based on the dsMTL^21^ framework and DataSHIELD [8]. In this Methods section, we first introduce the DataSHIELD environment. We then provide an overview of the intuition underlying dsLassoCov, followed by a formal definition of the optimization objective. Next, we describe the covariate adjustment mechanism and the associated optimization procedure, including the detailed algorithm. To maintain clarity, technical details are summarized here and provided in full in the Supplementary Methods, where additional information about the DataSHIELD infrastructure can also be found. Finally, we describe the simulation studies and real-data analyses used to evaluate the performance of dsLassoCov.

### DataSHIELD

DataSHIELD (ds) is a software created for data analysis at an individual level using disclosure-preventing methods that address ethical and legal restrictions concerning confidentiality^22,23^. Principal features of DataSHIELD involve: (I) a client-server architecture (“taking the analysis to the data”), (II) analytical methods for fedeated analysis including both mega and meta-analyses for the federated scenario, and (III) tailored multi-layer disclosure controls (the bottom line being that the analyst cannot see, copy or extract individual level data held by individual studies). The key aspects of DataSHIELD include its open-source nature, that is written in R, and is licensed under the GPL (General Public License), thus facilitating downstream analyses within a single pipeline by interacting with other programming languages (e.g., Python) and with other R or Bioconductor packages. The fundamental building blocks of DataSHIELD are its client-side and server-side functions. Server-side functions reside in the modified R environments located behind the firewall of the data computers at each individual study. It is the server-side functions that actually process the individual-level data at the distinct repositories. The outputs from server-side functions (non-disclosive study-level statistics) represent the only information that ever leaves a data computer, and this is why we can claim that DataSHIELD allows full analysis of individual-level data without those data ever having to be moved, or even rendered visible, outside their study of origin. Client-side functions reside on the conventional R environment on the analysis computer. Client-side functions call and control server-side functions and combine information across different repositories when required. All DataSHIELD functions require approval under a technical and governance process including external independent evaluation.

### Intuition behind dsLassoCov

A common approach to control confounding is regression adjustment, in which pre-specified covariates are included in the model. While effective in low-dimensional settings, this approach can become unstable in high-dimensional scenarios due to overfitting. Sparse regularization methods, such as LASSO, offer a natural alternative by selecting a subset of relevant predictors through penalization, thereby effectively creating a locally low-dimensional representation that is more suitable for covariate adjustment.

In dsLassoCov, control covariates are incorporated jointly with candidate features during model training but are excluded from penalization. As a result, features whose effects are explained by the covariates are less likely to be selected, and the estimated effects of the selected features are properly adjusted for confounding.

### Main model

1 $$\:\underset{w,u}{\mathrm{min}}\frac{1}{2n}{\left|\right|y-Xw-Cu\left|\right|}_{2}^{2}+\lambda\:{\left|w\right|}_{1}$$

Where, $\:X\in\:{\mathbb{R}}^{n\times\:p}$: feature matrix; $\:y\in\:{\mathbb{R}}^{n}$: outcome vector; $\:C\in\:{\mathbb{R}}^{n\times\:q}$ has full column rank: covariates matrix; $\:w\in\:{\mathbb{R}}^{p}$: coefficients of features; $\:u\in\:{\mathbb{R}}^{q}$: coefficients of covariates; $\:\lambda\:\ge\:0$: the regularization hyperparameter.

Formulation (1) refers to the proposed formulation of centralized dsLassoCov for a regression problem. $\:\left\{X,C,y\right\}$ refer to the feature matrix, covariates matrix and outcome vector of n samples. $\:\lambda\:{\left|w\right|}_{1}$aims to select important features. By solving the Formulation (1), we estimate the the pernalized coefficients $\:w$ and the unpenalized covariate coefficients $\:u$.

### Covariate adjustment interpretation

For any fixed $\:w$, the minimizer in $\:u$ is

2 $$\:{u}^{*}\left(w\right)={\left({C}^{T}C\right)}^{-1}{C}^{T}\left(y-Xw\right)$$

Substituting $\:{u}^{*}\left(w\right)$ into (1) yields the profiled objective,

3 $$\:\underset{w}{\mathrm{min}}\frac{1}{2n}{\left|\right|{M}_{C}\left(y-Xw\right)\left|\right|}_{2}^{2}+\lambda\:{\left|w\right|}_{1}$$

Where

$$\:{P}_{C}={\left({C}^{T}C\right)}^{-1}{C}^{T},\:{M}_{C}=I-{P}_{C}.$$

Defining the residualized quantities

$$\:\stackrel{\sim}{y}=\:{M}_{C}y,\:\stackrel{\sim}{X}=\:{M}_{C}X$$

The problem (3) becomes

4 $$\:\underset{w}{\mathrm{min}}\frac{1}{2n}{\left|\right|\stackrel{\sim}{y}-\stackrel{\sim}{X}w\left|\right|}_{2}^{2}+\lambda\:{\left|w\right|}_{1}$$

$\:{P}_{C}$ and $\:{M}_{C}$ refered to the column and null space of $\:C.$ Equation (4) shows that the regression estimator is obtained by applying the conventional Lasso to the components of $\:X$ and $\:y$ that are orthogonal to the column space of $\:C$. Therefore, dsLassoCov exactly adjusts for the part of the linear association that is explainable by the observed covariates $\:C$. This interpretation shows that dsLassoCov performs covariate adjustment in a way analogous to the classical partialling-out result (Frisch–Waugh–Lovell theorem), while simultaneously enabling sparse feature selection. A detailed proof can be found in the Proposition1 (Profiled objective) in the supplimentary method.

### The estimation of regulatrization path

Similar to other algorithms in dsMTL^21^, the $\:{\lambda\:}_{max}$ is estimated by applying KKT condition at $\:w=0$ on (4). A regularization path can then be constructed on a logarithmic grid from $\:{\lambda\:}_{max}$ down to its fraction. A detailed explaination can be found in the proposition 2 ($\:{\lambda\:}_{max}$) in the supplymentary method.

From (4), the KKT condition at $\:w=0$ is

$$\:\frac{1}{n}{\stackrel{\sim}{X}}^{T}\stackrel{\sim}{y}\in\:{\left.\lambda\:\partial\:{\left|w\right|}_{1}\right|}_{w=0}$$

Which is equivalent to

$$\:{||\frac{1}{n}{\stackrel{\sim}{X}}^{T}\stackrel{\sim}{y}||}_{\infty\:}\le\:\lambda\:$$

Hence the smallest value of λ for which the all-zero solution is optimal is

5 $$\:{\lambda\:}_{max}={||\frac{1}{n}{\stackrel{\sim}{X}}^{T}\stackrel{\sim}{y}||}_{\infty\:}=\underset{1\le\:j\le\:p}{\mathrm{max}}\left|\frac{1}{n}{{X}_{i}}^{T}{M}_{C}y\right|$$

### Optimization

Let

$$\:F\left(w,u\right)=\frac{1}{2n}{||y-Xw-Cu||}_{2}^{2},\:G\left(w\right)=\lambda\:||{w||}_{1}$$

The gradients of smooth part are

6 $$\:{\nabla\:}_{w}F\left(w,u\right)=\frac{1}{n}{X}^{T}\left(Xw+Cu-y\right){\nabla\:}_{u}F\left(w,u\right)=\frac{1}{n}{C}^{T}\left(Xw+Cu-y\right)$$

A proximal-gradient step with step size $\:t>0$ is

7 $$\:{u}^{k+1}={u}^{k}-t{\nabla\:}_{u}F\left({w}^{k},{u}^{k}\right)$$

8 $$\:{w}^{k+1}=\mathrm{S}\mathrm{o}\mathrm{f}\mathrm{t}\left({w}^{k}-t{\nabla\:}_{w}F\left({w}^{k},{u}^{k}\right),\:t\lambda\:\right)$$

where the soft-thresholding operator is applied elementwise:

$$\:{\mathrm{S}\mathrm{o}\mathrm{f}\mathrm{t}(z,\tau\:)}_{j}=\mathrm{s}\mathrm{i}\mathrm{g}\mathrm{n}\left({z}_{j}\right)\:\mathrm{m}\mathrm{a}\mathrm{x}\left(\left|{z}_{j}\right|-\tau\:,0\right)$$

Nesterov acceleration and backtracking line search has been used to improve convergence. The detailed algorithm can be found in the Supplementary Method.

### Federated implementation

Assume server $\:k\in\:\left[1,K\right]$ contained $\:{n}_{k}$ samples for federated analysis. The global loss and gradients become the sums of site-specific losses and gradients:

9 $$\begin{aligned}F\left(w,u\right)&=\sum\limits_{k=1}^{K}\frac{{n}_{k}}{n}{F}_{k}\left(w,u\right)\\ {\nabla\:}_{w}F(w,u)&=\sum\limits_{k=1}^{K}\frac{{n}_{k}}{n}{{\nabla\:}_{w}F}_{k}\left(w,u\right)\\ {\nabla\:}_{u}F(w,u)&=\sum\limits_{k=1}^{K}\frac{{n}_{k}}{n}{{\nabla\:}_{u}F}_{k}\left(w,u\right)\end{aligned}$$

Therefore, a client can obtain the exact centralized gradient by aggregating site-level non-disclosive quantities, so the federated optimization is algebraically equivalent to centralized optimization on pooled data, up to numerical tolerance. The detailed algorithm can be found in the supplementary method.

In contrast to centralized implementations such as glmnet, which rely on coordinate descent and update one coefficient at a time, dsLassoCov adopts a global proximal-gradient update tailored to the federated setting. At each iteration, the full parameter vectors $\:w$ and $\:u$ are updated simultaneously using aggregated site-specific gradient contributions as shown (7), (8) and (9).

This design is particularly advantageous in federated environments, where communication is often the primary bottleneck. While coordinate-wise updates would typically require frequent communication across sites, our approach requires only one round of communication per global iteration, with cost independent of the number of coefficients updated. This makes the method more suitable for high-dimensional federated applications.

### Simulation data analysis

The simulation analysis aims to evaluate the performance of dsLassoCov with respect to data dimensionality (efficiency), the number of servers (scalability), and the magnitude of confounding effects (robustness). In the simulated data, both the feature matrix and the outcomes are influenced by confounding effects as well as random noise. Therefore, a key objective is to assess how accurately the algorithm can reconstruct the ground-truth model (coefficient vector $\:{w}_{\mathrm{t}\mathrm{r}\mathrm{u}\mathrm{e}}$) under these conditions. To this end, three metrics were used to evaluate performance: runtime, feature selection accuracy, and coefficient estimation error.

Let $\:{\mathrm{S}}_{\mathrm{t}\mathrm{r}\mathrm{u}\mathrm{e}}$​ denote the set of true features. For each method, we obtain a coefficient vector $\:{w}_{estimate}$ (or feature importance scores for random forest). Features are ranked according to the absolute magnitude of their coefficients (or importance scores), and the top $\:\left|{\mathrm{S}}_{\mathrm{t}\mathrm{r}\mathrm{u}\mathrm{e}}\right|$ non-zero features are selected. The selected feature set is defined as:

$$\:{\mathrm{S}}_{\mathrm{s}\mathrm{e}\mathrm{l}\mathrm{e}\mathrm{c}\mathrm{t}\mathrm{i}\mathrm{o}\mathrm{n}}={\mathrm{T}\mathrm{o}\mathrm{p}}_{\left|{\mathrm{S}}_{\mathrm{t}\mathrm{r}\mathrm{u}\mathrm{e}}\right|}\left(\left|{w}_{estimate}\right|\right)$$

The feature selection accuracy is then defined as:

$$\:\mathrm{A}\mathrm{c}\mathrm{c}\mathrm{u}\mathrm{r}\mathrm{a}\mathrm{c}\mathrm{y}=\frac{\left|{\mathrm{S}}_{\mathrm{s}\mathrm{e}\mathrm{l}\mathrm{e}\mathrm{c}\mathrm{t}\mathrm{i}\mathrm{o}\mathrm{n}}\cap\:{\mathrm{S}}_{\mathrm{t}\mathrm{r}\mathrm{u}\mathrm{e}}\right|}{\left|{\mathrm{S}}_{\mathrm{t}\mathrm{r}\mathrm{u}\mathrm{e}}\right|}$$

For coefficient estimation error, the estimated coefficients $\:{w}_{estimate}$ are directly compared with the ground-truth coefficients $\:{w}_{\mathrm{t}\mathrm{r}\mathrm{u}\mathrm{e}},$

$$\:\mathrm{E}\mathrm{r}\mathrm{r}\mathrm{o}\mathrm{r}=\frac{{||{w}_{\mathrm{t}\mathrm{r}\mathrm{u}\mathrm{e}}-{w}_{estimate}||}_{2}^{2}}{\mathrm{l}\mathrm{e}\mathrm{n}\left({w}_{\mathrm{t}\mathrm{r}\mathrm{u}\mathrm{e}}\right)}$$

This metric is particularly informative, as it reflects the robustness of the method to confounding effects and noise, while also serving as a proxy for predictive performance. We do not report prediction performance directly, as the simulated outcomes are themselves confounded.

dsLassoCov was compared against two types of approaches: a federated baseline method and a meta-analysis of local machine-learning models. The federated baseline (denoted as “ds.glm + ds.LASSO”) consists of a two-step procedure. First, covariate effects are removed from both features and outcomes in a federated manner using ds.glm (generalized linear models), where one model is fitted per feature. Subsequently, a standard federated LASSO model (ds.LASSO) is trained on the adjusted data, incorporating all features into a single model for feature selection. For the meta-analysis approach, four base machine-learning models were considered: LASSO, ridge regression, support vector machines (SVM), and random forest. These models were trained locally on each server, and feature importance was aggregated by averaging the absolute values of the estimated coefficients (or importance scores) across sites. Prior to model training, covariates were regressed out from both features and outcomes separately at each site to control for confounding effects.

To ensure generalizability, both classification and regression tasks were simulated in all analyses. For classification tasks, however, covariate adjustment was applied only to the features and not to the outcome, due to the binary nature of the response variable. A detailed description of the machine learning workflow and cross-validation procedures used in the simulation analyses is provided in the Supplementary Methods.

For all simulation analysis, one confounding factor, 20 true features and 10 covariates are set. Each experiment is repeated 30 times to quantify the standard error. The detailed data generation process is described in the Supplementary Methods.

To justify the design of the simulation data generation process, we conducted an additional analysis to examine the structure of covariates and true signals in the simulated setting. The results demonstrate that our simulation captures a realistic and challenging scenario, characterized by strong spurious signals and weak true effects—conditions commonly encountered in high-dimensional biomedical data. This setting underscores the importance of jointly performing covariate adjustment and feature selection, as implemented in dsLassoCov. Detailed results are provided in the Supplementary Methods.

### Real data analysis

**Fig. 1:**
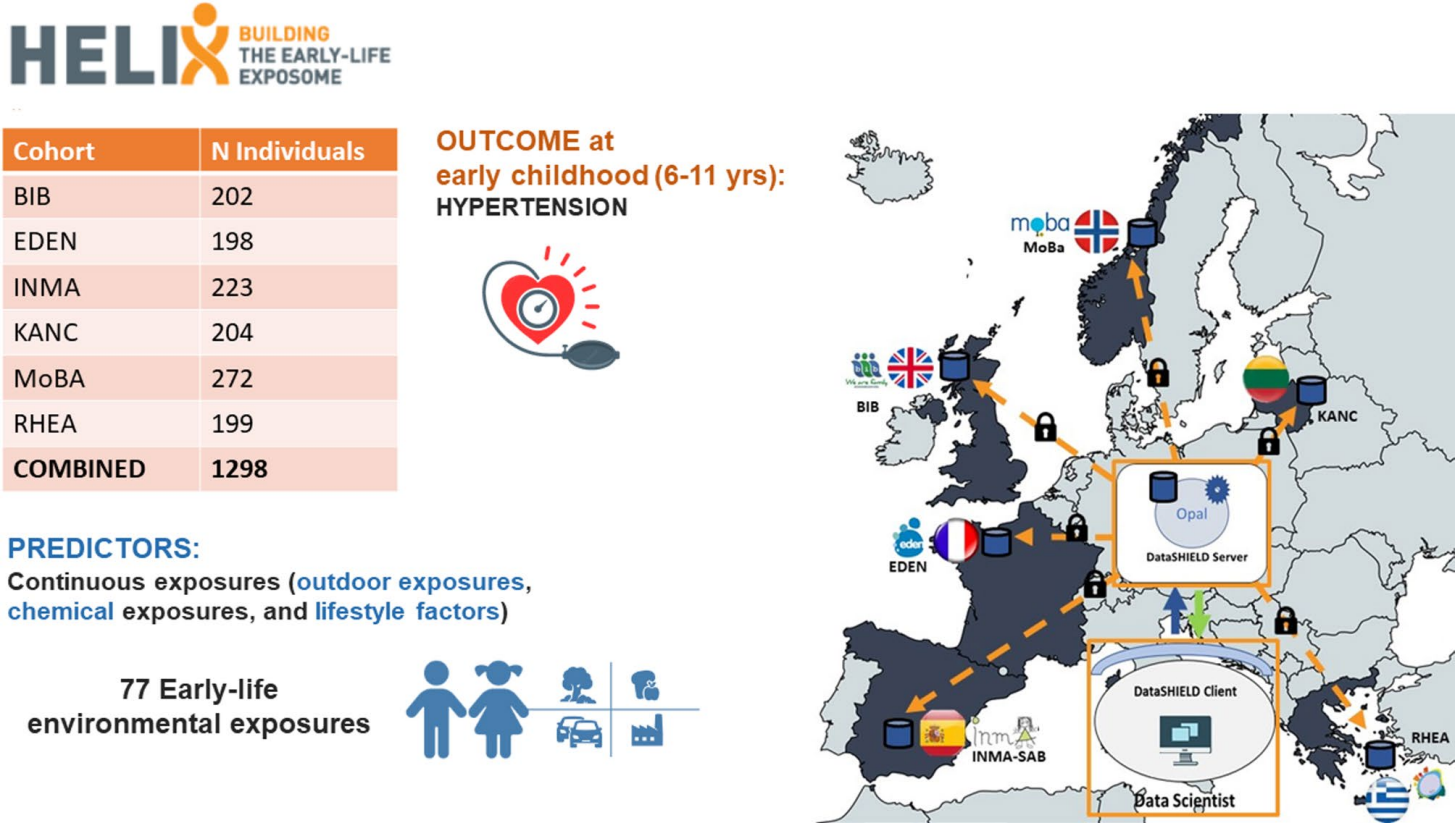
Input HELIX datasets organized by cohort in the Opal server.

This analysis is supported by the HELIX Project^20^, which investigates early life environmental risk factors impacting lifelong health issues. As illustrated in Fig. 1, data from the HELIX project are stored in six geographically distinct databases. A real DataSHIELD infrastructure, with each dataset hosted in a physically independent server, has been established among these databases to illustrate the usability of dsLassoCov in a real-world scenario.

The primary objective of this illustrative analysis was to identify early exposures associated with the diagnosis of hypertension. Early exposures in this context refer to environmental factors during pregnancy and the post-natal period, considered separately in the subsequent analyses. Two independent analyses were conducted, each involving 1143 healthy children and 155 children diagnosed with hypertension, distributed across the six data servers. In total, 77 exposures were included in the analysis, as detailed in Table S2. Additionally, 13 confounders which could potentially be associated with both the environmental exposures and blood pressure outcome were identified a priori based on previous literature^25,26^, and considered in the analysis. Further information regarding the HELIX project, data cohorts, data preprocessing steps, and covariate inference can be found in the supplementary methods.

For each analysis (e.g., “pregnancy” or “post-natal”), we initially applied dsLassoCov under a 5-fold cross-validation procedure over a sequence of 100 lambda values to tune the hyperparameter. Subsequently, a final dsLassoCov model is trained on all data cohorts using the selected hyperparameter.

## Results

### Simulation data analysis

In general, dsLassoCov consistently outperformed other approaches across all metrics for classification tasks. Regarding regression tasks, dsLassoCov demonstrated superior computational efficiency and indistinguishable feature selection and coefficient estimation ability compared to the federated baseline, and outperformed meta-analysis approaches.

#### Efficiency assessment

**Fig. 2:**
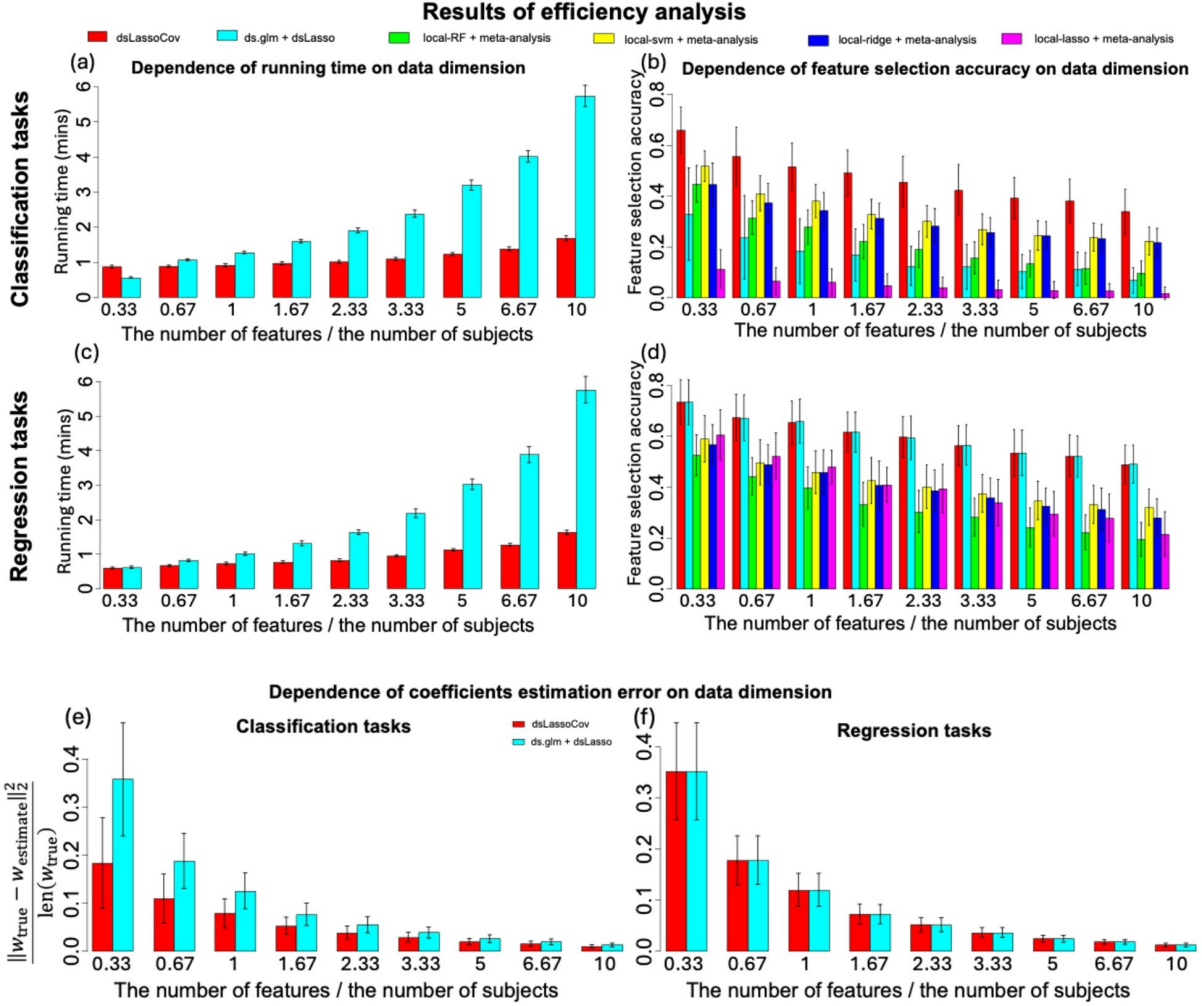
Result of the efficiency analysis. The dependence of algorithm performance on data dimensionality is evaluated for both regression and classification tasks. Performance is assessed using runtime, feature selection accuracy, and coefficient estimation error. Three servers were simulated, each containing 100 subjects. The number of features increased from 100 to 3000, corresponding to a feature-to-subject ratio ($\:\frac{\mathrm{F}\mathrm{e}\mathrm{a}\mathrm{t}\mathrm{u}\mathrm{r}\mathrm{e}{\mathrm{s}}^{{\prime\:}}\mathrm{n}\mathrm{u}\mathrm{m}\mathrm{b}\mathrm{e}\mathrm{r}}{\mathrm{S}\mathrm{u}\mathrm{b}\mathrm{j}\mathrm{e}\mathrm{c}\mathrm{t}{\mathrm{s}}^{{\prime\:}}\mathrm{n}\mathrm{u}\mathrm{m}\mathrm{b}\mathrm{e}\mathrm{r}}$) ranging from 0.33 to 10. The entire analysis is repeated 30 times to quantify the standard error. Panels (**a**), (**b**), and (**e**) present results for classification tasks, while panels (**c**), (**d**), and (**f**) correspond to regression tasks. Panels (**a**) and (c) show that dsLassoCov is more computationally efficient than the federated baseline, particularly in high-dimensional settings. As shown in panels (**b**) and (**d**), meta-analysis of local machine-learning models yields suboptimal feature selection accuracy. In classification tasks, dsLassoCov outperforms all competing approaches, whereas in regression tasks it achieves performance comparable to the federated baseline. A similar pattern is observed in panels (**e**) and (**f**): dsLassoCov more accurately reconstructs the ground-truth model than the federated baseline in classification settings, while showing comparable performance in regression settings. This imply that dsLassoCov produces more accurate and less confounding-driven model prediction, particularly in classification scenarios.

Figure 2 illustrates the results of the efficiency analysis comparing dsLassoCov against the federated baseline and meta-analysis approaches. For scenarios with low dimensionality (e.g., $\:\frac{\mathrm{F}\mathrm{e}\mathrm{a}\mathrm{t}\mathrm{u}\mathrm{r}\mathrm{e}{\mathrm{s}}^{{\prime\:}}\mathrm{n}\mathrm{u}\mathrm{m}\mathrm{b}\mathrm{e}\mathrm{r}}{\mathrm{S}\mathrm{u}\mathrm{b}\mathrm{j}\mathrm{e}\mathrm{c}\mathrm{t}{\mathrm{s}}^{{\prime\:}}\mathrm{n}\mathrm{u}\mathrm{m}\mathrm{b}\mathrm{e}\mathrm{r}}=0.33$), the run-time of dsLassoCov is comparable to the federated baseline (ds.glm+ds.Lasso). However, as data dimensionality increases, dsLassoCov exhibits gradually superior efficiency, as observed in Fig. 2a and c. Importantly, this superior performance is not attributed to a reduction in model ability.

Figures 2b and d highlight the increasing challenge of feature selection across all methods when the number of features in the dataset increases. Despite this challenge, dsLassoCov achieved higher (in classification settings) or indistinguishable (in regression settings) performance compared to other approaches. Specifically, in classification settings, dsLassoCov outperformed all other approaches, with some meta-analysis approach (e.g., SVM) following closely behind. In regression settings, as expected, dsLassoCov and “ds.glm + ds.lasso” performed comparably and better than meta-analysis approaches. Notably, compared to Fig. 2b, the results in (d) demonstrate that federated approaches in regression settings were more robust against higher dimensionality than in classification problems. For instance, for the regression task, an accuracy of approximately 60% was still achieved when $\:\frac{\mathrm{F}\mathrm{e}\mathrm{a}\mathrm{t}\mathrm{u}\mathrm{r}\mathrm{e}{\mathrm{s}}^{{\prime\:}}\mathrm{n}\mathrm{u}\mathrm{m}\mathrm{b}\mathrm{e}\mathrm{r}}{\mathrm{S}\mathrm{u}\mathrm{b}\mathrm{j}\mathrm{e}\mathrm{c}\mathrm{t}{\mathrm{s}}^{{\prime\:}}\mathrm{n}\mathrm{u}\mathrm{m}\mathrm{b}\mathrm{e}\mathrm{r}}$ was equal to 10, while in classification it barely reached an of accuracy of 30%. This discrepancy can be attributed to two factors: first, continuous outcomes in regression provide richer information for model estimation; second, in classification settings, confounding effects cannot be fully removed from binary outcomes through residualization, which limits the effectiveness of all approaches relying on this step.

Figures 2e and f report the coefficient estimation error, reflecting the ability to reconstruct the ground-truth model. A similar pattern is observed: dsLassoCov achieves more accurate reconstruction than the federated baseline in classification settings, while showing comparable performance in regression settings. These results further indicate that dsLassoCov produces more accurate and less confounding-driven prediction, particularly in classification scenarios.

#### Scalability assessment

**Fig. 3:**
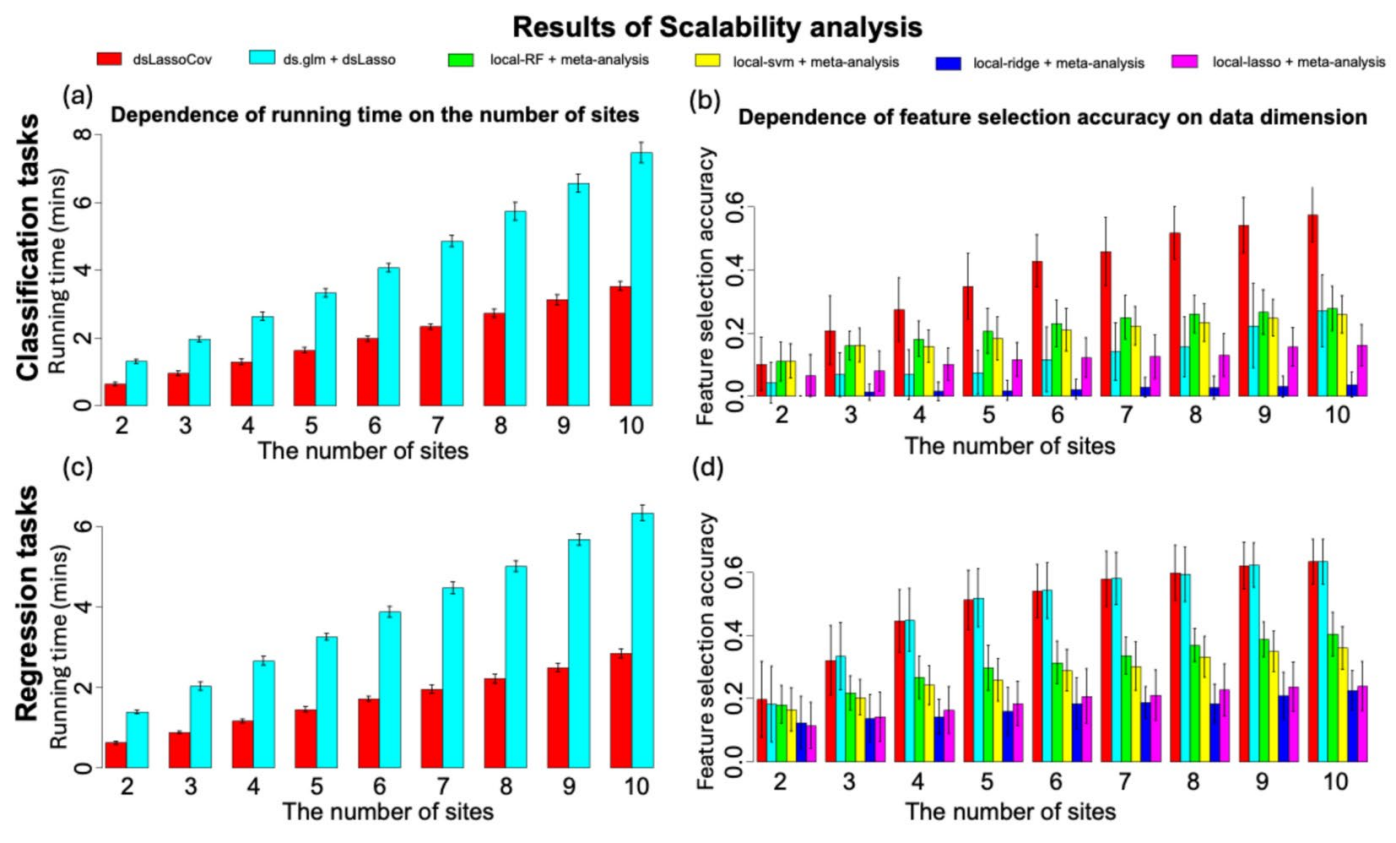
Results of the scalability analysis. We evaluated the dependence of algorithm performance on the number of participating sites under both classification and regression settings. Two metrics were considered: run time and feature selection accuracy. In this simulation, up to 10 sites were included, each containing 50 subjects and 1000 features. The number of sites was progressively increased, and the corresponding run time and feature selection accuracy were recorded. All tests are repeated 30 times to quantify the standard error of the metrics. Panels (**a**) and (**c**) present the run time comparisons across different federated approaches for classification and regression tasks, respectively. The results demonstrate that dsLassoCov is consistently more computationally efficient than conventional federated methods. Feature selection accuracy is shown in panels (**b**) and (**d**). In the classification setting (panel **b**), dsLassoCov achieves superior accuracy compared to all competing methods. In the regression setting (panel **d**), dsLassoCov shows comparable performance to conventional federated approaches, while maintaining its efficiency advantage.

Figure 3 presents the results of the scalability analysis. In Fig. 3a and c, the run-time of dsLassoCov and the conventional approach “ds.glm + ds.Lasso” is depicted with an increasing number of servers. Notably, dsLassoCov outperformed “ds.glm + ds.Lasso” for any given number of servers. Figure 3b and d illustrate the comparison of feature selection accuracy on classification and regression tasks, respectively as the number of servers increases. Firstly, we can observe how all approaches achieve higher performance with an increasing number of servers. Secondly, in the classification setting, we can notice how dsLassoCov outperforms all other approaches for any number of servers. In the regression setting, dsLassoCov performed comparably to “ds.glm + ds.Lasso” and outperformed all meta-analysis approaches.

#### Robustness assessment

**Fig. 4:**
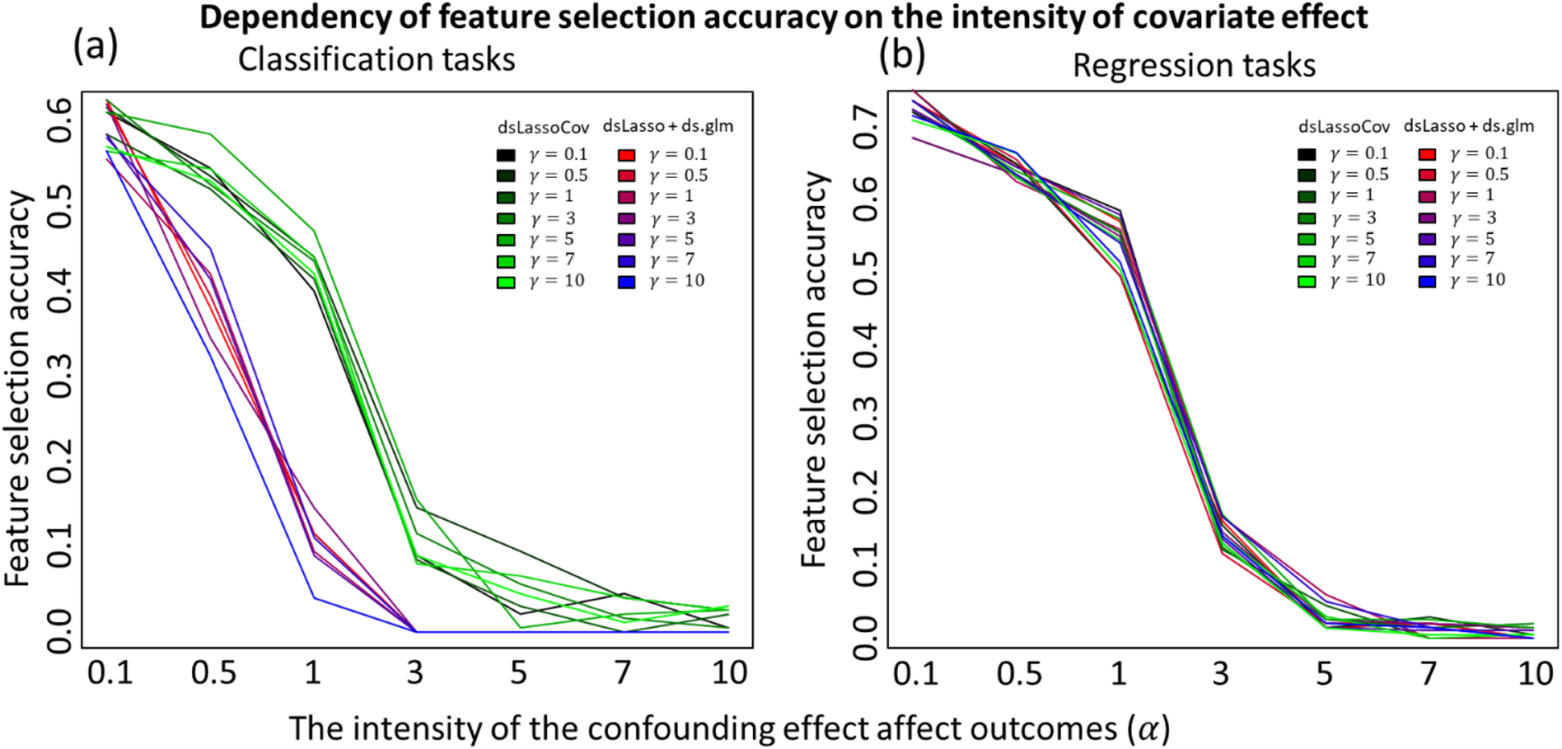
Result of the robustness analysis. The dependence of the feature selection accuracy on the intensity of confounding effects was explored in classification and regression setting. $\:\alpha\:$ and $\:\gamma\:$ referred to the intensities of the confounding effects affecting on outcomes and features, respectively. As the comparison, the conventional federated approach has been applied in the same setting. Three servers are simulated, and each contained 100 subjects with 1000 features. The parameter pair $\:\left\{\alpha\:,\gamma\:\right\}$ is varied from 0.1 to 10, and the simulated data is created for each variation. Panel (**a**) and (**b**) shows the results of classification and regression tasks. dsLassoCov outperformed the conventional federated approach in the classification setting, and performed indiscriminately in the regression setting. Moreover, compared to $\:\gamma\:$, $\:\alpha\:$ determines more the influence to feature selection accuracy.

Figure 4 presents the comparison of feature selection ability across different intensities of the confounding effect, denoted by α and γ, affecting outcomes and features, respectively. Consistent with previous findings, dsLassoCov outperforms “ds.glm + ds.Lasso” on classification tasks and exhibits comparable performance on regression tasks. Interestingly, it is observed that α has a stronger association with feature selection accuracy compared to γ. This observation can be attributed to the huge distorter effect that α elicits on associations in the simulated data. That is to say, our simulated outcomes comprises three effects: the true effect, the confounding effect, and an additional independent random effect. In scenarios with a high proportion of the confounding effect (high α value), the true effect is diminished, while the random effect remains unaffected. Consequently, the random effect becomes relatively amplified, posing a more challenging problem for feature selection.

### Real data analysis

**Fig. 5:**
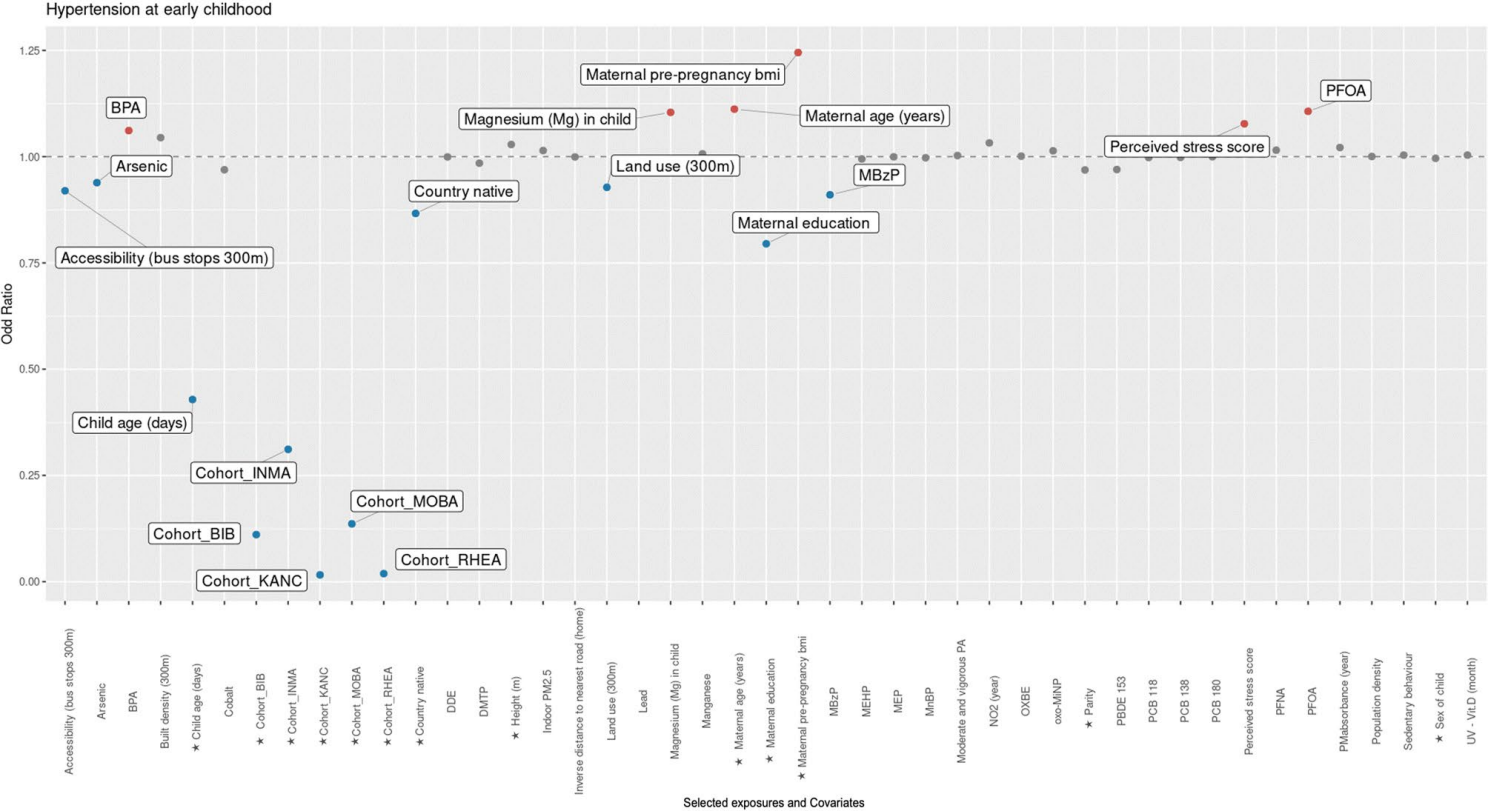
Estimated odd ratios for selected exposures in the real HELIX dataset (Model: HT ~ exposome + confounders). Only exposures showing an odd ratio higher than 1.05 or lower than 0.95 are plotted. Confounders are identified here with an astherics (*). Red indicates a positive odd ratio, while blue indicates a negative odd ratio.

To demonstrate the applicability and usability of our dsLassoCov approach, we decided to conduct a real-world data analysis, involving a real dataset under a real DataSHIELD infrastructure. We leveraged the HELIX exposome dataset, which comprises one of the largest exposome resources for the pediatric population (~ 8 years old). Data from six independent cohorts (each dataset hosted in a different dataSHIELD server) were analyzed. To recap, our research question aimed to identify early-life environmental exposures associated with hypertension. The dsLassoCov model selected 32 exposures with an odd ratio distinct from (OR < 0.95 or > 1.05) for hypertension risk, as detailed in Table S3 and illustrated in Fig. 5. Among these selected exposures, the half were identified as risk factors for hypertension, encompassing outdoor exposures (such as building density), chemical exposures (such as PFAS), and lifestyle factors (such as exposure to a high-stress environment). The other half were highlighted as protective factors for hypertension and involved urban exposures such as the presence of public transport near to the area of residence. Notably, these findings replicate insights previously described^25,26^, thereby reinforcing the reliability and trustability of dsLassoCov.

To investigate the comparability of our federated approach against the local traditional LASSO approach, we conducted LASSO analysis in a non-federated manner by merging data from all cohorts on the same computer and running the local-dsLassoCov. In this scenario, the reported optimal lambda value slightly differed (0.009) compared to federated dsLassoCov, resulting in a more sparse model. This discrepancy may be attributed to the optimization method employed by local traditional dsLassoCov, which favors selecting highly sparse solutions through coordinate descent^24^. However, this optimization approach is not feasible for federated learning adaptation due to its feature-wise nature, resulting in computationally intractable communication costs. Despite the different number of exposures selected, those chosen by local-dsLassoCov exhibited roughly the same estimated coefficients as those by federated dsLassoCov, as indicated in Table S3. Additionally, there were 13 exposures selected by federated dsLassoCov but ignored by local-dsLassoCov, with odds ratios close to 1. This minor discrepancy is deemed reasonable and may be attributed to sampling variation during the cross-validation procedure.

## Discussion

In this manuscript, we introduce dsLassoCov - a federated learning algorithm designed to efficiently and effectively control confounding effects in high-dimensional settings. We first provided a detailed exposition of the techniques underpinning dsLassoCov, including insights into its modeling, optimization, model training, and the federated implementation. Second, we offered a theoretical interpretation of the covariate control mechanism within dsLassoCov, illustrating how it implicitly control the effects of covariates. Third, our simulation data analysis revealed that dsLassoCov showed significantly higher or equal performane compared to other approaches in terms of speed, feature selection accuracy and coefficient estimation error. Lastly, in our real data analysis, we showcase dsLassoCov’s efficacy in capturing hypertension-associated early exposures, aligning well with previous research findings^25,26^.

These results support the utility of dsLassoCov for biomedical applications, particularly in exposome studies where large datasets from multiple population cohorts are available. Recent trends in the field emphasize the importance of protecting sensitive patient data, as exemplified by initiatives like HELIX^20^, ATHLETE^27^ or LifeCycle projects^23^, which serve as pioneering consortia for implementing federated analysis in epidemiological studies. In this context, dsLassoCov provides a federated learning solution for two major challenges encountered in exposome analysis: (1) high data dimensionality and (2) the presence of strong confounding effects and multicollinearity phenomena. Failing to address these challenges properly can lead to reduced power in detecting true associations and a higher likelihood of biased results, such as overestimating the effect of one exposure while underestimating another. dsLassoCov can address both situations and may therefore be a useful tool for effectively leveraging the resources generated by these and future consortia. By offering a robust solution to these challenges, dsLassoCov facilitates more accurate and reliable analysis of exposome data, ultimately advancing our understanding of environmental exposures and their impact on health outcomes.

Despite its utility, dsLassoCov does have limitations. First, as demonstrated in the simulation studies for regression tasks, dsLassoCov achieves feature selection and coefficeint estimation performance comparable to the ds.glm + ds.Lasso approach, indicating similar robustness to confounding effects. It is worth noting that the ds.glm + ds.Lasso pipeline is a widely adopted strategy for covariate adjustment in biomedical research, including molecular^28^, exposome^25^, clinical^29^ studies. In this regard, dsLassoCov may serve as a computationally more efficient alternative within federated learning settings, as suggested by its superior efficiency observed in our simulations. Secondly, the current formulation is the assumption that the covariate matrix $\:C$ has full column rank, ensuring that $\:{C}^{T}C$ is invertible. While this assumption is standard in low-dimensional adjustment settings, it may be violated in the presence of collinearity or high-dimensional covariates. In such cases, a stabilized formulation can be adopted by replacing the inverse with $\:{\left({C}^{T}C+\rho\:I\right)}^{-1}$, corresponding to an L2-regularized estimate of the covariate effects. Extending the method to this more general setting is a direction for future work. Thirdly, dsLassoCov is integrated into the DataSHIELD^8^ infrastructure, which currently supports only cross-silos federated learning applications, thereby limiting its usage in cross-device federated learning applications. Addressing this limitation would require further development in collaboration with DataSHIELD to expand into cross-device^6^ federated learning applications.

Regarding future development perspectives, it is worth noting that the current dsLassoCov implmentation is primarily designed for prediction and sparse feature selection, and does not provide formal guarantees for false discovery rate (FDR) control, which is often critical in biomedical research. A promising direction is to develop a dsLassoCov-based workflow that enables statistical inference on the selected features. For example, dsLassoCov could be used as a feature screening step, followed by federated statistical modeling (e.g., generalized linear models within the DataSHIELD framework) to obtain p-values and estimate the FDR. To ensure valid inference, feature selection and downstream inference should be performed on independent samples, which can be achieved in federated settings through in-site sample partitioning at each participating site.

## Conclusion

We introduce dsLassoCov, a federated learning method designed to perform LASSO regression while addressing confounding effects. Positioned as a novel approach in the realm of federated learning, dsLassoCov stands out as a specialized solution tailored to combat a significant source of heterogeneity: confounding effects prevalent in various biomedical applications. With its unique focus on confounding effects, dsLassoCov has the potential to serve as a foundational element in transitioning numerous biomedical studies into the realm of large-scale data-driven analyses.

## Supplementary Information

Below is the link to the electronic supplementary material.

Supplementary Material 1

## Data Availability

Tutorial: https://rpubs.com/aanguita/986397. For the simulation analysis, data generation details are provided in our GitHub repository listed above. For the real data analysis, data access requests should be directed to the corresponding author, Xavier Basagaña, at [xavier.basagana@isglobal.org](mailto: xavier.basagana@isglobal.org) .

## Code availability

Software: The software used was dsMTLClient 0.9.9 and dsMTLBase 0.9.9. The client-side package (version of the package that needs to be installed in the analyst computer): https://github.com/transbioZI/dsMTLClient. The server-side package (version of the package that needs to be installed in the analyst computer): https://github.com/transbioZI/dsMTLBase.
