# Supplementary material for: dsLassoCov: a federated Lasso approach incorporating covariate control
Source: Sci Rep. 2026 Apr 22;16:18759. doi: 10.1038/s41598-026-48845-0 (PMC13272686; doi:10.1038/s41598-026-48845-0)
Supplement: Supplementary file 1 — Supplementary Material 1 [file 41598_2026_48845_MOESM1_ESM.pdf]

Supplementary Material of “dsLassoCov: a federated Lasso approach incorporating covariate control”

Outlines:

- Modelling, optimization, training protocol and algorithm
    - Main model
    - Profiling out covariate effects (Proposition 1)
    - Optimality conditions and regularization path (Proposition 2)
    - Optimization via Proximal Gradient
    - Training and regularization path
    - The federated implementation
    - Federated learning algorithm
- Simulation data analysis
    - The competing methods
    - Machine learnming workflow
    - 5 fold Cross-validation
    - Confounded data simulation
    - Efficiency assessment
    - Scalability assessment
    - Robustness assessment
    - Structure of covariates and true signals
- Real data analysis
    - Helix project
    - Data cohorts and preprocessing
    - Covariate inference
- Introduction of DataSHIELD
- Supplementary Tables
- Supplementary Figures

Modelling, optimization and algorithm

Main Model

Let's consider the linear prediction model f(x, w) = xw, the loss function becomes,

$$\min_{w,u} \frac{1}{2n} ||y - Xw - Cu||_2^2 + \lambda|w|_1 \tag{S1}$$

Where,
$X \in \mathbb{R}^{n \times p}$: feature matrix
$y \in \mathbb{R}^n$: outcome vector
$C \in \mathbb{R}^{n \times q}$ has full column rank: covariates matrix
$w \in \mathbb{R}^p$: coefficients of features
$u \in \mathbb{R}^q$: coefficients of covariates
$\lambda \geq 0$: the regularization hyperparameter.

Formulation (1) refers to the proposed formulation of centralized dsLassoCov for a regression problem. $\{X, C, y\}$ refer to the feature matrix, covariates matrix and outcome vector of n samples. $\lambda|w|_1$ aims to select important features. By solving the Formulation (1), we estimate the the pernalized coefficients w and the unpenalized covariate coefficients u.

Profiling out covariate effects

Assumption 1 (Full column rank)

We assume that C has full column rank, i.e. $\text{rank}(C) = q$, *so that $C^T C$ is invertible.*

Proposition 1 (Profiled objective)

For any fixed w, the minimizer in u is

$$u^*(w) = \left(C^T C\right)^{-1} C^T (y - Xw) \tag{S2}$$

Substituting $u^*(w)$ into (S1) yields the profiled objective,

$$\min_{w} \frac{1}{2n} ||M_C(y - Xw)||_2^2 + \lambda|w|_1 \tag{S3}$$

Where

$$P_C = (C^T C)^{-1} C^T, M_C = I - P_C$$

Proof

For fixed w, (S1) is quadratic in u. Taking derivative:

$$\nabla_u L(w, u) = \frac{1}{n} C^T (Xw + Cu - y)$$

Setting to zero gives:

$$C^T C u = C^T (y - Xw),$$

which yields (S2). Substituting into the residual:

$$y - Xw - Cu^* = (I - P_C)(y - Xw) = M_C(y - Xw)$$

Residualized formulation

Define:

$$\tilde{y} = M_C y, \tilde{X} = M_C X$$

Then (S3) becomes

$$\min_w \frac{1}{2n} ||\tilde{y} - \tilde{X}w||_2^2 + \lambda |w|_1 \tag{S4}$$

Interpretation

Equation (S4) shows that dsLassoCov is equivalent to performing Lasso on variables that are orthogonal to the column space of covariates C. Thus, the method removes linear effects of covariates before feature selection.

Optimality conditions and regularization path

Proposition 2 (λ_{max})

Under KKT condition, the optimal solution w^*satisfies:

$$0 \in \frac{1}{n} \tilde{X}^T (\tilde{X}w^* - \tilde{y}) + \lambda \partial \|w^*\|_1 \tag{S5}$$

The smallest λ such that $w = 0$ is optimal is:

$$\lambda_{max} = \left\| \frac{1}{n} \tilde{X}^T \tilde{y} \right\|_\infty = \max_{1 \le j \le p} \left| \frac{1}{n} X_i{}^T M_C y \right| \tag{S6}$$

Proof

Evaluate (S5) at $w = 0$:

$$\frac{1}{n} \tilde{X}^T \tilde{y} \in \lambda[-1,1]$$

Thus:

$$\left\| \frac{1}{n} \tilde{X}^T \tilde{y} \right\|_\infty < \lambda$$

Optimization via Proximal Gradient

Define smooth part:

$$F(w,u) = \frac{1}{2n}\|y - Xw - Cu\|_2^2,$$

Gradients:

$$\nabla_w F(w,u) = \frac{1}{n}X^T(Xw + Cu - y),\ \nabla_u F(w,u) = \frac{1}{n}C^T(Xw + Cu - y)$$

Since F is smooth and convex, and $\lambda|w|_1$ is convex but non-smooth, we adopt a proximal gradient method.

At iteration k, we update:

$$w^{k+1} = \mathrm{prox}_{\lambda/L}\left(w^k - \frac{1}{L}\nabla_w F(w^k, u^k)\right) \quad \text{(S7)}$$

$$u^{k+1} = u^k - \frac{1}{L}\nabla_u F(w^k, u^k) \quad \text{(S8)}$$

where L is the Lipschitz constant.

Closed-form Updates

Covariates (no penalty)

$$u^{k+1} = u^k - \frac{1}{L}\nabla_u F(w^k, u^k)$$

Features (L1 penalty → soft-thresholding)

$$w^{k+1} = Soft\left(w^k - \frac{1}{L}\nabla_w F(w^k, u^k), \frac{\lambda}{L}\right)$$

where the soft-thresholding operator is applied elementwise:

$$\mathrm{Soft}(z,\tau)_j = \mathrm{sign}(z_j)\max(|z_j| - \tau, 0)$$

Nesterov acceleration

We apply Nesterov acceleration[1-3]:

$$\mathrm{s}^k = \frac{\alpha_{k-1}}{\alpha_k} w^k + \frac{1-\alpha_{k-1}}{\alpha_k} \mathrm{w}_{k-1},$$

and performs the update at s^k.

Training and regularization path

Instead of estimating a model for each specific λ (lambda), we derived the entire regularization path across a sequence of λ values, organized in descending order. This sequence represents a range of potential hyperparameter values, where a higher λ is associated with fewer identified features. A sequence of M λ is created,

$$\lambda_1 = \lambda_{max} > \cdots > \lambda_M$$

where:

$$\lambda_{max} = \max_{1 \le j \le p} \left| \frac{1}{n} {X_i}^T M_C y \right|$$

as shown in (S6)

To establish this optimal sequence, we utilized a strategy inspired by the glmnet[4] procedure. The smallest λ in the sequence is then set relative to λ_max (e.g., λ_max*0.01), with the complete sequence being interpolated on a logarithmic scale. The "warm-start" technique detailed in our prior work[5] is used for training.

The federated implementation

Our federated algorithm leverages the DataSHIELD[6] infrastructure, wherein data are securely stored on DataSHIELD servers and interact exclusively with the DataSHIELD Client.

Assume server $t \in [1, T]$ contained n_t samples for federated analysis. For the dsLassoCov algorithm, two essential functions are deployed on the server-side: one to access the gradient of the loss function$\nabla_w F_t(w,u)$ and $\nabla_u F_t(w,u)$ using the local data of n_t samples, and another to evaluate the loss function $F_t(w,u)$. These functions are invoked during runtime, with their results transmitted back to the client. On the client side, values from all servers are aggregated to ascertain the final gradient and function value, as outlined below.

$$F(w,u) = \sum_{t=1}^{T} \frac{t}{n} F_t(w,u) \qquad \text{(S9)}$$

$$\nabla_w F(w,u) = \sum_{t=1}^{T} \frac{n_t}{n} \nabla_w F_t(w,u) \qquad \text{(S10)}$$

$$\nabla_u F(w,u) = \sum_{t=1}^{T} \frac{n_t}{n} \nabla_u F_t(w,u) \qquad \text{(S11)}$$

Federated learning algorithm

Algorithm 1 summarizes the algorithm performed on the client side to solve the objective, wherein functions (S9~S11) required the remote access to the servers and aggregated the resturns. The estimation of the regularization path is summarized in Algorithm 2. Prior to Algorithm 2, the lambda sequence is estimated as shown above.

Algorithm 1 dsLassoCov client-side algorithm

Input: $\lambda > 0$, $L_0 > 0$, w^0, u^0, $maxIter > 0$

Output: w^{i+1}, u^{i+1}

1: Initialize $w^1 = w^0$, $u^1 = u^0$, $\alpha_{-1} = \alpha_0 = 0$, and $L = L_0$

2: **for** $i = 1$ to $maxIter$ **do**

3: Set $S^i = w^i + \frac{\alpha_{i-1}-1}{\alpha_i}(w^i - w^{i-1})$

4: Set $S^i{}_u = u^i + \frac{\alpha_{i-1}-1}{\alpha_i}(u^i - u^{i-1})$

5: **for** L in $\{L_{i-1}, 2L_{i-1}, 4L_{i-1}, 16L_{i-1}, \ldots\}$ **do**

6: $G_w = \nabla_w F(S^i, S^i{}_u)$ according to (S10)

7: $G_u = \nabla_u F(S^i, S^i{}_u)$ according to (S11)

8: Update w^{i+1} according to (S7)

9: Update u^{i+1} according to (S8)

10: $\mathcal{M} = F(w^i, u^i) + \langle G_w, w^{i+1} - w^i \rangle + \langle G_u, u^{i+1} - u^i \rangle + \frac{L}{2}\left|\left|w^{i+1} - w^i\right|\right|_2^2 + \frac{L}{2}\left|\left|u^{i+1} - u^i\right|\right|_2^2$

11: **if** $\mathcal{M} \geq F(w^{i+1}, u^{i+1})$ according to (S9) **do**

Break

end if

end for

12: Set $L_i = L$, and $\alpha_{i+1} = \frac{1+\sqrt{1+4\alpha_i^2}}{2}$

13: If termination rule satisfied, **return**

end for

Algorithm 2 Estimation procedure of regularization path

Input: $\lambda_1 > \lambda_2 > \cdots > 0$

Output: $\{w_0, u_0\}$, $\{w_1, w^{(c)}{}_1\}$, $\{w_2, u_2\}$,…

1: Initialize $w_0 = 0$, $u_0 = 0$,

2: **for** $i = \{1, 2, \ldots\}$ **do**

3: W_i =**Algorithm 1** ($\lambda = \lambda_i$, $L_0 = 1$, $w_0 = w_{i-1}$, $u_0 = u_{i-1}$, $maxIter = 100$)

Simulation data analysis

We conducted a simulation-based data analysis to demonstrate the efficacy, scalability, and robustness of our novel method. Efficiency analysis is focused on quantifying the algorithm's performance as the data dimensionality increases. Scalability analysis, on the other hand, aims to evaluate how the algorithm performs as the number of servers utilized grows. Lastly, robustness analysis delves into understanding how varying degrees of confounding effects impact the algorithm's effectiveness. The primary metrics employed for evaluating the algorithm's utility include runtime, feature selection accuracy and coefficeints estimation error.

The competing methods

To facilitate a comprehensive comparison of runtime, we employ the conventional federated approach, termed "ds.glm + dsLasso", as our baseline. This approach involves two main steps: first, utilizing ds.glm() from the dsBaseClient[7] package to execute a federated generalized linear model, which aims to mitigate covariate effects from the features and outcomes (known as “regressing out covariates”[8] approach). Subsequently, dsLasso() from the dsMTL[5] package is employed to train a federated Lasso model. The simulation of servers is executed on a single machine using the dsLite[7] package.

For assessing feature selection accuracy and coefficeints estimation error, the "ds.glm + dsLasso" and meta-analysis are used as a comparison. In the meta-analysis, four distinct machine learning models—namely, Random Forest (RF), Support Vector Machine (SVM), Lasso, and Ridge Regression—are trained locally on each server.

Machine learnming workflow

We perform a 5-fold cross-validation first to select the hyperparameters (if there are any) and then a model training to obtain the coefficients. For feature selection accuracy, the following calculation is performed.

Let $\mathrm{S_{true}}$ denote the set of true features in the simulation and w the obtained coefficient vector $w_{estimate}$ (or feature importance scores for random forest). Features are ranked according to the absolute magnitude of their coefficients (or importance scores), and the top $|\mathrm{S_{true}}|$ non-zero features are selected.

We define the selected feature set as:

$$\mathrm{S_{selection}} = \mathrm{Top}_{|\mathrm{S_{true}}|}(|w_{estimate}|)$$

The feature selection accuracy is then defined as:

$$\mathrm{Accuracy} = \frac{|\mathrm{S_{selection}} \cap \mathrm{S_{true}}|}{|\mathrm{S_{true}}|}$$

For dsLassoCov and "ds.glm + dsLasso", the coefficients w can be read from the learnt model. For the meta-analysis, the site-specific w_t is learnt for each site t. These scores are averaged among T sites to form the final score $\frac{1}{T}\sum_{t=1}^{T}|w_t|$.

For coefficient estimation error, the estimated coefficients $w_{estimate}$ are directly compared with the ground-truth coefficients w_{true},

$$\text{Error} = \frac{\|w_{\text{true}} - w_{estimate}\|_2^2}{\text{len}(w_{\text{true}})}$$

5 fold Cross-validation

The cross-validation is performed within each server site (in-site cross-validation) rather than across sites. Specifically, for each site, samples are randomly partitioned into 5 folds locally. During each cross-validation iteration, the training and test splits are generated independently at each site without sharing individual-level data across sites.

For a given fold, the model is trained using the training subsets from all sites via the federated optimization procedure, while evaluation is performed on the corresponding local test subsets at each site. The performance metrics (mean squared error for regression or misclassification rate for classification) are first computed locally and then aggregated across sites using a sample-size-weighted average:

$$\text{Error} = \sum_{t=1}^{T} \frac{n_t}{\sum_{t'} n_{t'}} \text{Error}^{(t)}$$

where n_t denotes the number of samples at site t.

Confounded data simulation

To evaluate the ability of dsLassoCov to recover true signals under confounding, we simulate data where a latent confounder simultaneously affects both the outcome and a subset of features. Without proper adjustment, this induces spurious associations between non-causal features and the outcome.

Simulation Setup

We generate data with the following parameters:

- n: number of subjects
- p: total number of features
- p_t: number of true (causal) features
- p_c:number of confounded (non-causal) features
- α: strength of confounder on the outcome

- γ: strength of confounder on features

Confounder Generation

A latent confounder $c \in \mathbb{R}^n$ is generated as:

$$c_i = \text{sign}(z_i) \times u_i, z_i \sim N(0,1), u_i, \sim N(1,1)$$

This produces a symmetric distribution with non-zero magnitude.

Feature Matrix Construction

We generate the design matrix:

$$X = \left[X_c, X_{sig}, X_{non}\right] \in \mathbb{R}^{n \times p}$$

where:

- $X_{sig} \in \mathbb{R}^{n \times p_t}$: true features
- $X_c \in \mathbb{R}^{n \times p_c}$: confounded features
- $X_{non} \in \mathbb{R}^{n \times (p - p_c - p_t)}$: noise features

All entries are initially sampled as:

$$X \sim N(0,1)$$

Injecting Confounding into Features

Each confounded feature is influenced by the latent confounder:

$$X_c^{(j)} \leftarrow X_c^{(j)} + \gamma c, \;\; j = 1, \dots, p_c$$

This induces correlation between X_c and c, while keeping them non-causal.

Afterward, all features are standardized:

$$X_{sig}, X_c \text{ are column-wise z-normalized}$$

True Signal Generation

The coefficients for true features are sampled as:

$$w_i = \text{sign}(z_i) \times u_i, z_i \sim N(0,1), u_i, \sim N(1,1)$$

with $w \in \mathbb{R}^{p_t}$.

Outcome Generation

The outcome is generated as:

$$Y = X_{sig}w + \alpha c$$

Then, Y is standardized.

Observation Noise and Task Type

Regression setting:

$$Y \leftarrow Y + \varepsilon, \varepsilon \sim N(0,0.5)$$

Classification setting:

$$Y \leftarrow \text{sign}(Y + \varepsilon), \varepsilon \sim N(0,0.1)$$

Efficiency assessment

In our efficiency test, our objective was to measure how the runtime of the algorithm correlates with the increasing dimensionality of the data. The above simulation process is applied. We simulated three servers, each housing the feature data of 100 'subjects' along with their corresponding outcomes, which could be binary or continuous. Among these features, 20 were genuinely associated with the outcomes. Additionally, we introduced a single confounding effect that influenced 10 spuriously outcome-associated features. The intensity parameters governing the confounding effects on both outcomes and features were set to 1. We systematically varied the feature dimension, selecting values sequentially from the set $\{100, 200, 300, 500, 700, 1000, 1500, 2000, 3000\}$. The parameters γ and α were fixed at 1 for this experiment.

Scalability assessment

In our scalability test, our objective was to assess how the runtime of the algorithm varies with an increasing number of servers. A total of 10 servers were simulated for this experiment. Each server housed the feature data of 50 independent 'subjects' along with their corresponding outcomes, which could be binary or continuous. The feature dimension was fixed at 1000, with 20 features truly significant to the outcomes. Additionally, we introduced a single confounding effect that influenced 10 spuriously outcome-associated features. The parameters γ and α were set to 1 for this experiment. This setup allowed us to systematically explore the impact of scaling the number of servers on algorithm runtime.

Robustness assessment

In our robustness test, our goal was to assess how the intensity of the confounding effect impacts the algorithm's feature selection capability. To conduct this analysis, we generated feature data and outcomes

(which could be binary or continuous) for 300 'subjects'. These subjects were evenly distributed across three simulated servers, with each subject possessing 1000 features.

We systematically varied the parameters α and γ, where $\alpha \in \{0.1, 0.5, 1, 3, 5, 7, 10\}$ and $\gamma \in \{0.1, 0.5, 1, 3, 5, 7, 10\}$, representing the intensity of the confounding effects affecting on the coutcome and features. For every combination of these two parameters, we trained both the dsLassoCov and "ds.glm + dsLasso" algorithms. Subsequently, we compared the resulting feature selection accuracy. This comprehensive examination allowed us to evaluate the algorithm's robustness across various levels of confounding effects.

Structure of covariates and true signals

Under the classification setting, the simulation parameters are summarized in Table S4. The data generation process follows the same configuration described in the previous section.

To illustrate the roles of the confounder and true signals, we conducted a series of logistic regression analyses and computed the corresponding odds ratios (ORs) under different model specifications:

The model specification	Interpratation
y ~ confounder	Association between the confounder and the outcome
y ~ signal	Marginal association of true signal features
y ~ signal + confounder	Adjusted association of true signals
y ~ null	Marginal association of confounded (non-causal) features
y ~ null + confounder	Adjusted association of confounded features

In the simulation, 20 true signal features and 10 confounded (null) features were generated. For each setting, we report the average OR across features. All experiments were repeated 30 times with different random seeds.

Interpretation of Results

As shown in **Figure S2**, the confounder exhibits a strong association with the outcome. Confounded (non-causal) features also display strong marginal associations, despite having no direct effect on the outcome. This demonstrates the classical confounding effect, where shared dependence on a latent variable induces spurious associations.

In contrast, individual true signal features show relatively weak marginal associations. However, their effects become a little more interpretable after adjusting for the confounder, indicating that their contributions are partially masked by confounding.

Importantly, once the confounder is included in the model, the apparent association of confounded features disappears, confirming that these associations are entirely driven by confounding rather than causal effects.

These results highlight two key challenges:

- **Spurious signals:** Confounded features may appear strongly associated with the outcome if covariates are not controlled.
- **Weak true signals:** Individual causal features may exhibit only modest marginal effects.

Together, this motivates the need for methods such as dsLassoCov, which simultaneously perform covariate adjustment and feature selection, enabling the recovery of true signals under confounding.

Read data analysis

To showcase the practical utility of dsLassoCov in real-world multi-center studies, we opted for complex exposome data. Our focus was on utilizing dsLassoCov to identify early environmental exposures that potentially influence the risk of hypertension.

Helix project

The data cohorts investigated here are derived from the HELIX (Human Early-Life Exposome) Project[9]. The HELIX project gathers data from 6 longitudinal-based European birth cohorts with the aim of evaluating the effect of environmental risk factors on the health of mothers and children. We used data from the Helix sub-cohort consisting of data from approximately 200 children in each cohort (Table S1). In the 1,298 children, a wide range of environmental exposures were evaluated to define the early-life exposome during two time periods: the prenatal pregnancy period and the postnatal period (childhood age 6 to 11 years). Exposures were estimated based on the home and school address (provided details here on in the supplement). A general description of the study population can be found in the Supplementary **Table S1**, and a summary of the structure and organization of the dataset in the federated scenario is illustrated in **Figure 2**.

The data cohorts under investigation stem from the HELIX (Human Early-Life Exposome) Project. This initiative aggregates data from six longitudinal-based European birth cohorts with the overarching objective of assessing the impact of environmental risk factors on the health outcomes of both mothers and children. Specifically, we utilized data from the Helix sub-cohort, which comprises approximately 200 children from each cohort (refer to **Table S1** for details). Within a cohort of 1,298 children, an extensive array of environmental exposures was scrutinized to delineate the early-life exposome across two distinct time periods: the prenatal pregnancy period and the postnatal period spanning childhood ages 6 to 11 years. These exposures were assessed based on information obtained from both home and school addresses (further elaborated in the supplement). For a comprehensive understanding of the study population, please refer to **Table S1**. Furthermore, **Figure 2** provides a succinct depiction of the structure and organization of the dataset within the federated scenario.

Data cohorts and preprocessing

Environmental exposures used in this analysis consisted of outdoor exposures, chemical exposures and lifestyle factors. Outdoor exposures included built environment, meteorological conditions, natural

spaces, road traffic, and noise. Chemical exposures included: organochlorine compounds (including polychlorinated biphenyls [PCBs] and organochlorine pesticides), polybrominated diphenyl ethers, per- and polyfluoroalkyl substances (PFAS), metals, phthalate metabolites, phenols, organophosphate pesticide metabolites, and cotinine). Lifestyle factors included smoking habits, physical activity, allergens, sleep, and socioeconomic status. Details on the correlations between assessed exposures has been previously documented 19 Additional information regarding the type of assessed exposures can be found in the Supplementary Table S2. Informed consent was available for the use of the data as described in this manuscript and ethics approval for such use was available.

The environmental exposures considered in this analysis encompassed outdoor factors, chemical constituents, and lifestyle determinants. Outdoor exposures were categorized into built environment parameters, meteorological conditions, natural surroundings, road traffic influences, and noise levels. Chemical exposures included a broad spectrum of substances such as organochlorine compounds (including polychlorinated biphenyls [PCBs] and organochlorine pesticides), polybrominated diphenyl ethers, per- and polyfluoroalkyl substances (PFAS), various metals, phthalate metabolites, phenols, organophosphate pesticide metabolites, and cotinine. Lifestyle factors examined encompassed smoking habits, levels of physical activity, exposure to allergens, sleep patterns, and socioeconomic status. For further details on the correlations between these assessed exposures, readers are referred to previous documentation[10]. Additional information pertaining to the types of exposures assessed can be found in **Table S2**. Informed consent was obtained for the usage of the data as outlined in this manuscript, and ethical approval for such usage was obtained. The outcome variable was represented as a binary variable indicating the diagnosis of hypertension, in accordance with the 2004 reference charts from the National High Blood Pressure Education Program. The outcome has been previously investigated in this population, and the main findings of the research can be found in[11,12].

In total, 1143 healthy and 155 children diag nosed with hypertension are included in the analysis. 77 early exposures are included as the features, together with inferred 13 covariates. Prior to analysis, all variables included in the dataset underwent appropriate preprocessing steps, which involved the removal of outliers, normalization and standardization of features, and imputation of missing values, as detailed in previous literature[18].

Covariate inference

Exposure data was available for both the prenatal and postnatal periods, necessitating the execution of separate models for each time point. To account for potential confounding factors, a Directed Acyclic Graph (depicted in **Figure S1**) was utilized, identifying 13 variables as potential confounders. These variables included factors from both the prenatal and postnatal periods.

The confounding variables encompassed the cohort of recruitment, maternal age (continuous in years), maternal educational level (categorized as low, middle, or high), self-reported maternal pre-pregnancy body mass index (continuous in kg/m2), parity (classified as nulliparous, primiparous, or multiparous), native status (indicating whether the child's family is native to the country of recruitment), as well as the child's age, height, and sex. Cohort of recruitment was represented by six dummy variables, one for each population involved, with only five of them being included as confounders in the analysis. This approach ensured proper adjustment for potential biases arising from cohort differences.

Introduction of DataSHIELD

Whilst several tools and platforms have recently become available in federated learning[13], the so-called 'DataSHIELD' infrastructure has gained considerable attention in the epidemiological and biomedical fields[14]. DataSHIELD is an R-based software solution for individual level federated analysis using disclosure-preventing methods to ensure ethico-legal compliance[6,15]. Principal features of DataSHIELD involve: I) a client-server architecture, II) a bunth of builtin and community-developed federated packages[16] for, e.g., federated visualization as well as omics analysis, and III) tailored multi-layer disclosure controls (to ensure that the analyst cannot see, copy or extract individual level data held by individual studies). The key advantages of DataSHIELD include its open-source nature, that is written in R, and is licensed under the GPL, thus facilitating downstream analyses within a single pipeline by interacting with other programming languages (e.g., Python) and with other R or Bioconductor packages.

DataSHIELD is currently used within a number of H2020 and Horizon Europe projects (ref LifeCycle, ATHLETE). Whilst a wide range of statistical methods have been implemented within DataSHIELD, there is currently a lack of state-of-the-art machine learning (ML) approaches commonly used in biomedicine and epidemiology[5,7]. Machine learning techniques include methods for variable selection, aimed at identifying the 'best' subset of features for predicting a given study outcome or phenotype. These techniques can increase the predictive capacity and interpretability of models while tackling overfitting when applied to high-dimensional data, such as those often found in the exposome and omics. A key method currently previously unavailable in DataSHIELD is Lasso regression – a regularization technique similar to ordinary least squares, except that it imposes a shrinkage penalty on the magnitude of the estimated coefficients[17,18]. Lasso is among the most straightforward and frequently employed feature-selection techniques[4], specially for the analysis of complex epidemiological datasets[19]. In the current paper, we describe some of the challenges of implementing LASSO in a federated setting, and present 'dsLassoCov', an implementation within DataSHIELD of the Lasso feature-selection method.

1. Nesterov, Y. Gradient methods for minimizing composite functions. *Mathematical Programming* **140**, 125-161 (2012).
2. Beck, A. & Teboulle, M. A fast iterative shrinkage-thresholding algorithm for linear inverse problems. *SIAM journal on imaging sciences* **2**, 183-202 (2009).
3. Liu, J. & Jieping, Y. Efficient L1/Lq Norm Regularization.
4. Zou, H. & Hastie, T. Regularization and variable selection via the elastic net. *Journal of the Royal Statistical Society: Series B (Statistical Methodology)* **67**, 301-320 (2005).
5. Cao, H. *et al.* dsMTL - a computational framework for privacy-preserving, distributed multi-task machine learning. *Bioinformatics* (2022).
6. Gaye, A. *et al.* DataSHIELD: taking the analysis to the data, not the data to the analysis. *International journal of epidemiology* **43**, 1929-1944 (2014).
7. Marcon, Y. *et al.* Orchestrating privacy-protected big data analyses of data from different resources with R and DataSHIELD. *PLoS Comput Biol* **17**, e1008880 (2021).

8. Snoek, L., Miletić, S. & Scholte, H.S. How to control for confounds in decoding analyses of neuroimaging data. *Neuroimage* **184**, 741-760 (2019).
9. Maitre, L. *et al.* Human Early Life Exposome (HELIX) study: a European population-based exposome cohort. *BMJ Open* **8**, e021311 (2018).
10. Tamayo-Uria, I. *et al.* The early-life exposome: description and patterns in six European countries. *Environment international* **123**, 189-200 (2019).
11. Warembourg, C. *et al.* Early-Life Environmental Exposures and Blood Pressure in Children. *J Am Coll Cardiol* **74**, 1317-1328 (2019).
12. Warembourg, C. *et al.* Urban environment during early-life and blood pressure in young children. *Environ Int* **146**, 106174 (2021).
13. Mullie, L. *et al.* CODA: an open-source platform for federated analysis and machine learning on distributed healthcare data. *Journal of the American Medical Informatics Association* **31**, 651-665 (2024).
14. Wolfson, M. *et al.* DataSHIELD: resolving a conflict in contemporary bioscience—performing a pooled analysis of individual-level data without sharing the data. *International journal of epidemiology* **39**, 1372-1382 (2010).
15. Jaddoe, V.W.V. *et al.* The LifeCycle Project-EU Child Cohort Network: a federated analysis infrastructure and harmonized data of more than 250,000 children and parents. *Eur J Epidemiol* **35**, 709-724 (2020).
16. community, D. Packages developed in DataSHIELD.
17. Tibshirani, R. Regression shrinkage and selection via the lasso: a retrospective. *Journal of the Royal Statistical Society: Series B (Statistical Methodology)* **73**, 273-282 (2011).
18. Tibshirani, R. Regression shrinkage and selection via the lasso. *Journal of the Royal Statistical Society. Series B (Methodological)*, 267-288 (1996).
19. Santos, S. *et al.* Applying the exposome concept in birth cohort research: a review of statistical approaches. *European journal of epidemiology* **35**, 193-204 (2020).

Supplementary Tables

Table S1 – Description of the study population

Variable name	N (%)	Min	Q1	Median	Q3	Max
Maternal age at birth	1298 (100 %)	16	27.3	31	34.	43.5
Pre-pregnancy BMI	1298 (100 %)	15.9	21.3	23.8	27.1	51.4
Child age at the follow up	1298 (100 %)	5.4	6.5	8	8.9	12.1
SBP (mmHg)	1298 (100 %)	70.5	92	98.5	106.5	159
DBP (mmHg)	1298 (100 %)	37	52	57	62.5	118.5

Variable name	N (%)	Category	n (%)
Hypertension	1298 (100 %)	Control	1143 (88.1 %)
		Case	155 (11.9 %)
Cohort	1298 (100 %)	BiB	202 (15.6%)
		EDEN	198 (15.2%)
		INMA	223 (17.2%)
		KANC	204 (15.7%)
		MoBA	272 (21%)

		RHEA	199 (15.3%)
Highest maternal education	1298 (100 %)	Primary	180 (13.9%)
		Secondary	444 (34.2%)
		Higher	674 (51.9%)
Child sex	1298 (100 %)	Girl	590 (45.5%)
		Boy	708 (54.5%)

Population: study population from the HELIX subcohort, n=1298 children.
Acronyms: DBP, Diastolic Blood Pressure, SBP: Systolic Blood Pressure, BiB: Born in Bradford, EDEN: Étude des Déterminants pré et postnatals du développement et de la santé de l'Enfant, INMA: Infancia y Medio Ambiente, IOTF: International Obesity Task Force, KANC: Kaunus Cohort, MoBa: The Norwegian Mother, Father and Child Cohort Study, RHEA: Mother-Child Cohort in Crete.

Table S2 – List of all exposures measured and studied

Type of exposure	Exposures	Number of exposures Childhood
**URBAN EXPOSOME**		
**Outdoor air pollution**	NO_2, PM_{10}. $PM_{2.5}$, $PM_{2.5}$ absorbance	4
**Indoor air pollution**	NO_2, $PM_{2.5}$, PM absorbance, Benzene	4
**Meteorology**	Temperature, UV	2
**Surrounding natural spaces**	NDVI	1
**Built environment**	Accessibility, population density, building density, street connectivity, accessibility, facility richness, walkability, land use index	8
**Road traffic and noise**	Traffic load on all roads and nearest road, traffic density on nearest road, inverse distance to nearest road.	3
Total of urban exposures for each period		22
**LIFESTYLE**		

**Diet**	KIDMED score	1
**Physical activity**	Moderate/vigorous activity, sedentary time	2
**Socio-economic & others**	House crowding, self-perceived stress score	2
Total of lifestyle exposures for each group		5
**CHEMICAL EXPOSOME**		
**Organochlorine pesticides (OCs)**	PCB, DDE, DDT, HCB	8
**Perfluoroalkyl substances (PFASs)**	PFHxS, PFOS, PFOA, PFNA, PFUnDA	5
**Brominated compounds (PBDEs)**	PBDE 47, 153	2
**Metal and essential elements**	As, Hg, Cd, Pb, Cs, Cu, Mn, Co, Mo, Se, Tl	14
**Phthalates**	MECPP, MEHHP,MEHP, MEOHP , MEP , MiBP, MnBP, MBzP, DEHP [a], oxo-MiNP and oh-MiNP	10
**Phenols**	MEPA, ETPA, PRPA, BUPA, BPA, OXBE, TRCS	7
**Organophosphate (OP) metabolites**	DMP, DMTP, DEP, DETP, DMDTP	4
Total of chemical exposures for each group		50
**Total of exposures**		**77**

[a] DEHP: molar sum of MEHP, MEHHP, MEOHP and MECPP.

Table S3. Estimated Odd ratios in the real HELIX dataset (Model: HT ~ exposome + confounders).

**Feature**	**OR (dsLassoCov)**	**OR (local-dsLassoCov)**	**Group**
Cohort_KANC	**0.016**	**0.012**	Confounder
Cohort_RHEA	**0.019**	**0.012**	Confounder
Cohort_BIB	**0.111**	**0.119**	Confounder
Cohort_MOBA	**0.136**	**0.139**	Confounder
Cohort_INMA	**0.311**	**0.370**	Confounder
Child age (days)	**0.429**	**0.414**	Confounder
Maternal education	**0.795**	**0.802**	Confounder
Country native	**0.866**	**0.877**	Confounder
MBzP	**0.910**	**0.935**	Phthalates

Accessibility (bus stops 300m)	**0.920**	**0.869**	Built Environment
Land use (300m)	**0.928**	**0.941**	Built Environment
Arsenic	**0.939**	**0.950**	Metals
Parity	**0.969**	**0.986**	Confounder
Cobalt	0.969	0.000	Metals
PBDE 153	**0.970**	**0.989**	PBDEs
Lead	0.978	0.000	Metals
DMTP	0.985	0.000	OP Pesticides
MEHP	0.994	0.000	Phthalates
Sex of child	**0.996**	**1.003**	Confounder
MnBP	0.997	0.000	Phthalates
PCB 118	0.998	0.000	OCs
PCB 138	0.998	0.000	OCs
Inverse distance to nearest road (home)	0.999	0.000	Traffic
DDE	0.999	0.000	OCs
MEP	0.999	0.000	Phthalates
PCB 180	0.999	0.000	OCs
Population density	1.0003	0.000	Built Environment
OXBE	1.001	0.000	Phenols
Moderate and vigorous PA	1.003	0.000	Lifestyle
Sedentary behaviour	1.004	0.000	Lifestyle
UV - Vit.D (month)	1.004	0.000	Meteorological
Manganese	1.007	0.000	Metals
oxo-MiNP	1.014	0.000	Phthalates
Indoor PM2.5	1.014	0.000	Indoor air
PFNA	1.015	0.000	PFASs
PMabsorbance (year)	1.021	0.000	Air Pollution
Height (m)	**1.029**	**1.013**	Confounder

NO2 (year)	1.032	0.000	Air Pollution
Built density (300m)	**1.045**	**1.030**	Built Environment
BPA	**1.061**	**1.042**	Phenols
Perceived stress score	**1.077**	**1.068**	Others
Magnesium (Mg) in child	**1.104**	**1.082**	Essential minerals
PFOA	**1.107**	**1.082**	PFASs
Maternal age (years)	**1.112**	**1.090**	Confounder
Maternal pre-pregnancy bmi	**1.245**	**1.249**	Confounder

Confounders were forced to retain in the final output model in both dslassoCov and local-dslassoCov. Features are in increasing order according to the estimated odd ratio. Odd ratios in bold correspond to features selected in both dsLassoCov and local-dslassoCov models. Underlined features correspond to exposures selected by the two models that are not confounders.

Table S4: the configuration of parameters used to create simulation datasets

	value	interpretation
α	1	The intensity of confounding effect affacting on outcome
γ	1	The intensity of confounding effect affacting on features
ε	$N(0,0.1)$	A random noise when generating the outcome
Confounder		One column vector indicating the confouding effect
$\text{null} \in X_c$	-	One of the 10 features that are affected by Confounder
$\text{signal} \in X_{sig}$	-	One of the 20 true features generating the outcome

Supplementary Figures

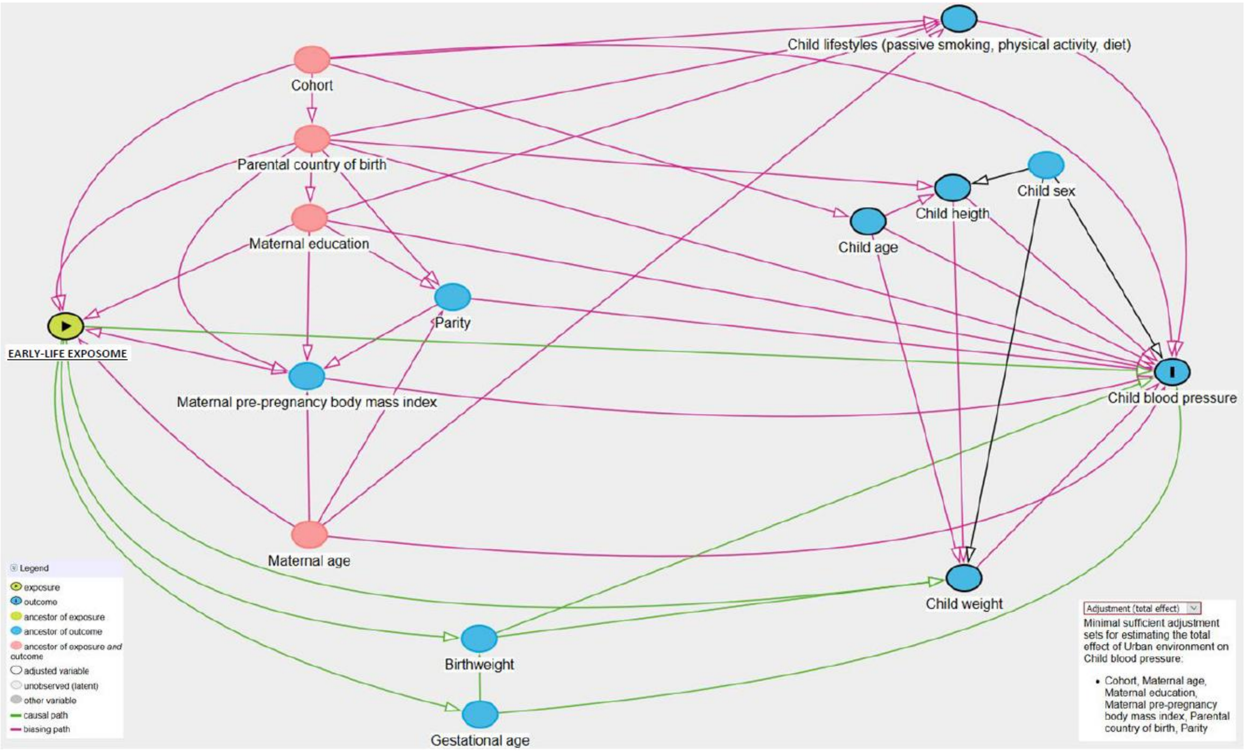

Figure S1, Direct Acyclic Graph design to identified confounder in the association between the early-life exposome and child blood pressure.

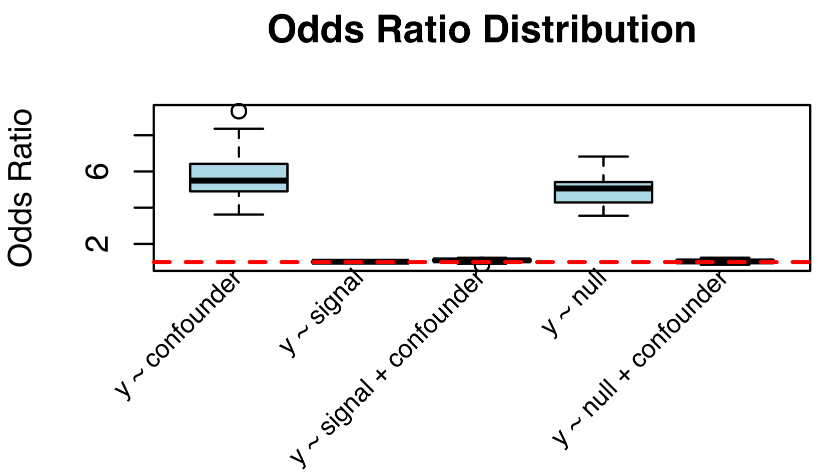

Figure S2, The structure of covariates in the simulation data
